# Exercise Training Enhances Myocardial Mitophagy and Improves Cardiac Function via Irisin/FNDC5-PINK1/Parkin Pathway in MI Mice

**DOI:** 10.3390/biomedicines9060701

**Published:** 2021-06-21

**Authors:** Hangzhuo Li, Shuguang Qin, Qiaoqin Liang, Yue Xi, Wenyan Bo, Mengxin Cai, Zhenjun Tian

**Affiliations:** Institute of Sports and Exercise Biology, School of Physical Education, Shaanxi Normal University, Xi’an 710119, China; lhz798627074@snnu.edu.cn (H.L.); sunnyqin3@gmail.com (S.Q.); liangqq@snnu.edu.cn (Q.L.); xiyue@snnu.edu.cn (Y.X.); boweny9909@snnu.edu.cn (W.B.); 2017mxc@snnu.edu.cn (M.C.)

**Keywords:** exercise training, myocardial infarction, mice, mitophagy, irisin

## Abstract

Myocardial infarction is the major cause of death in cardiovascular disease. In vitro and in vivo models are used to find the exercise mode which has the most significant effect on myocardial irisin/FNDC5 expression and illuminate the cardioprotective role and mechanisms of exercise-activated myocardial irisin/FNDC5-PINK1/Parkin-mediated mitophagy in myocardial infarction. The results indicated that expression of irisin/FNDC5 in myocardium could be up-regulated by different types of exercise and skeletal muscle electrical stimulation, which then promotes mitophagy and improves cardiac function and the effect of resistance exercise. Resistance exercise can improve cardiac function by activating the irisin/FNDC5-PINK1/Parkin-LC3/P62 pathway, regulating mitophagy and inhibiting oxidative stress. OPA1 may play an important role in the improvement of cardiac function and mitophagy pathway in myocardial infarction mice by irisin-mediated resistance exercise. Resistance exercise is expected to become an effective therapeutic way to promote myocardial infarction rehabilitation.

## 1. Introduction

Myocardial infarction (MI) is a major cause of death in cardiovascular diseases [1]. During MI, the level of local myocardial oxidative stress elevated abnormally due to ischemia and hypoxia, resulting in the death of a large number of cardiomyocytes, myocardial fibrosis and eventually heart failure [2]. Exercise can improve cardiac function, rescue necrotic myocardium [3,4] and reduce the incidence of cardiovascular events by regulating myocardial oxygenation and ventricular remodeling after MI [5]. Mitophagy can remove damaged and aging mitochondria and maintain cell homeostasis [6]. Oxidative stress leads to abnormal mitophagy and cardiac dysfunction [7], and its effective therapeutic target and mechanism need to be further studied.

Irisin is a secreted peptide proteolytically processed from transmembrane protein fibronectin type III domain protein 5 (FNDC5) [8]. It was originally found in mice and human serum and can be secreted in skeletal muscle, myocardium, adipose and other tissues [9]. Irisin can reduce heart injury caused by ischemia and hypoxia through regulating mitochondrial function [10]. During myocardial ischemia, hypoxia and abnormal energy metabolism as well as impaired cardiac function, mitophagy could improve cardiac function by removing damaged mitochondria [11]. PTEN-induced kinase 1/ E3 ubiquitin ligase parkin (PINK1/Parkin) is the main pathway of mitophagy, it can induce mitophagy through recruiting microtubule-associated protein light chain 3 (LC3II/I) and SQSTM1/p62 (P62) at the mitochondria [12]. Exercise-activated mitophagy was mediated by the irisin/FNDC5-PINK1/Parkin-LC3/P62 pathway, which could significantly increase the level of myocardial anti-oxidation and improve cardiac function [13]. As the myogenic exerkine [8], irisin could result in clearer cardioprotection by exercise training [14,15], However, whether this protective effect works through PINK1/Parkin-mediated mitophagy still lacks experimental evidence. Furthermore, whether the cardioprotective effect of exercise also works through this mechanism and which exercise mode is better in MI myocardium still remain to be studied. The purpose of this study is to sift out the most significant effect of exercise mode on myocardial irisin/FNDC5 expression and explore the mechanism of myocardial irisin/FNDC5-PINK1/Parkin-mediated mitophagy induced by exercise, which will provide experimental basis for the screening methods of cardiac rehabilitation after MI.

## 2. Materials and Methods

### 2.1. Animal and Exercise Protocol

Animal: Male C57BL/6J wildtype (WT) mice (8-weeks old) were purchased from the Laboratory of Animal Centre of Xi’an Jiaotong University (Xi’an, China). After one week of acclimatization, WT mice were randomly divided into 8 groups: control, aerobic exercise, resistance exercise, vibration exercise, electrical stimulation, sham, MI and MI resistance exercise (*n* = 6/group). The MI model was established by permanent ligation of the left anterior descending coronary artery (LAD) of the heart. (The preparation procedure of myocardial infarction model is as follows: Mice were anesthetized with isoflurane, the limbs were fixed on an operating table after skin preparation, the chest was opened and the heart was squeezed, and ligation was performed 1–2 mm below the left anterior descending coronary artery). The sham group underwent the same surgical procedure without ligation. The MI resistance exercise group received resistance exercise training one week after LAD recovery. All animal experiments were applied to the ARRIVE guidelines [16] and were approved by the ethical committee of Shaanxi Normal University (NO: 201916003, 7 July 2019).

**Figure 1:**
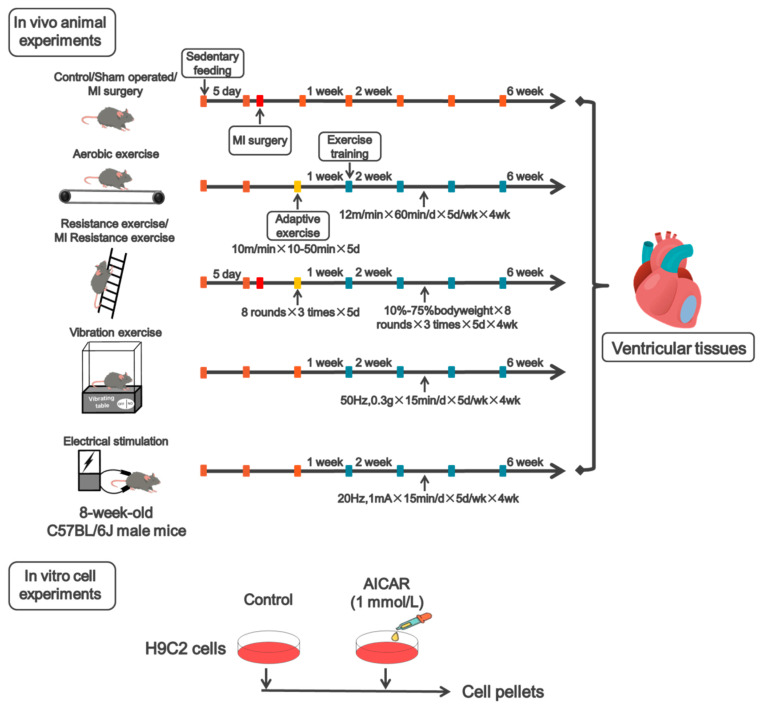
The schematic diagram of animal exercise protocol and cell intervention.

Exercise protocol: Aerobic exercise refers to previous experiments and the research of Sonobe et al. [17]. In the first week, adaptive treadmill training was performed. Mice were trained at 10 m/min for 10 min on the first day, and increased by 10 min per day, ending up to 10 m/min for 50 min on the fifth day. From the second week on, the exercise training was performed at 12 m/min for 60 min per day, 5 days per week for 4 weeks. Resistance exercise refers to previous experiments and the research of Horii et al. [18]. The ladder is 1.1 m high, separated by 1 cm, and inclined at 85°. In the first week, the mice underwent 1 week of no load-adaptive training. From the second week on, the mice underwent another 4 weeks of normal exercise with incremental load. The weight hung on the tails of the mice was increased from 0% to 75% of body weight, and was increased at a rate of 10% a day in the following days. The mice underwent 8 rounds of training per day, 3 times of climbing the ladder per round, 1 min rest between each round, lasting 5 days per week for 4 weeks. Vibration exercise refers to the research of Ren et al. [19]. Vibration platform provided vertical vibrations at 50 Hz with a peak-to-peak magnitude of 0.3 g, lasting 15 min per day, 5 days per week for 4 weeks. The Electrical stimulation protocol refers to the research of Su et al. [20]. The Electrical stimulation device delivered pulses at 20 Hz with 1 mA, lasting 15 min per day, 5 days per week for 4 weeks (Figure 1).

### 2.2. Cell Culture

H9C2 rat cardiomyoblasts were purchased from the Cell Culture Center of the Institute of Basic Medical Sciences, Chinese Academy of Medical Sciences, Beijing, China (item No.3111C0001CCC000219). H9C2 cells were cultured in Dulbecco’s modified eagle medium (DMEM) supplemented with 10% fetal calf serum, penicillin (100 U/mL), and streptomycin (10 µg/mL) (Gibco BRL, Grand Island, NE, USA) and were maintained at 37 °C in a 5% CO_2_ incubator (Thermo Model 371, Marietta, OH, USA). Cultured H9C2 cells were randomly divided into 2 groups: control group (Control), 5-amino-1-β-d-ribofuranosyl-imidazole-4-carboxamide (AICAR) and intervention group (AICAR; 1 mmol/L). AICAR is widely used as an activator of Adenosine monophosphate (AMP)-activated protein kinase (AMPK), and it simulates AMPK-related metabolic effects of exercise at the cellular level (Figure 1) [21]. Cell morphologic changes were observed under an inverted microscope (Leica DMIL LED, Jena, Germany).

### 2.3. Echocardiographic Measurements

To measure cardiac physiological functions, an ultrasound cardiotachograph (VINNO 6 VET, VINNO, Suzhou China) was used for the detection and analysis of echocardiography. After the exercise, the mice are anesthetized with isoflurane and fixed on the operating table after skin preparation. B-mode ultrasonography was applied in seeking the long axis of left ventricular, then 2-dimensional M-mode was transferred for data acquisition. Left ventricle internal dimension diastole (LVIDd), left ventricle internal dimension systole (LVIDs) and ejection fraction (EF) were directly measured by using the M-mode analysis. Fractional shortening (FS) was calculated by LVIDd and LVIDs (calculation formula: FS = (LVIDd − LVIDs)/LVIDd × 100%).

After 4 weeks of exercise training, the mice were anesthetized with isoflurane and fixed. Their heart was quickly collected with their chest opened in a low temperature environment and placed in liquid nitrogen for subsequent experiments. Heart tissue cryopreserved in liquid nitrogen is used for molecular testing.

### 2.4. Blood Collection and Biochemical Index Measurement

After the mice were anesthetized with isoflurane, blood sample were collected from the orbit and stored in a 37 °C water pot for 4 h, and then centrifuged at 1000 rpm for 1 min. After centrifugation, the upper serum was transferred to a new EP tube and stored at −20 °C for subsequent experiments. All steps were strictly performed in accordance with the kit instructions.

By using the assay kits (Beyotime, Shanghai, China), we detected the renal dysfunction biomarkers including serum Total Superoxide dismutase (T-SOD), Malondialdehyde (MDA). All steps were carried out strictly according to kit instructions.

### 2.5. Extraction and Detection of Mitochondrial Protein

The tissue mitochondrial protein extraction kit (BB-31711, BestBio, Shanghai, China) was used to extract mitochondrial protein from mice myocardium. The protein concentration was determined by the bicinchoninic acid protein quantitative method. The protein was denatured at 100 °C, then stored at −80 °C for follow-up Western Blotting experiment. All steps were carried out strictly according to kit instructions.

### 2.6. RT-qPCR

Total RNA was extracted from frozen heart tissues (100 mg) and H9C2 cells with Trizol reagent (Invitrogen, Sao Paulo, Brazil). RNA concentration was determined spectrophotometrically at 260/280 nm wavelength using a commercial kit (BioTek Epoch, Winooski, VT, USA). First-strand cDNA was generated from RNA using Revertaid First Strand cDNA synthesis kit (TaKaRa, Kusatsu, Japan). PCR reactions were performed with quantitative PCR instrument (CFX connectTM Real-Time system, Bio-Rad, Singapore) using SYBR Green/ROX qPCR Master Mix (TaKaRa, Kusatsu, Japan) and quantified using Bio-Rad CFX manager. GAPDH was used as the internal control gene. The primer sequences used for RT-qPCR analyses are shown as follows:

FNDC5(F:5′-GGCTGGGAGTTCATGTGGAA-3′; R:5′-TGGGAAGCGGTTATCTTTGCT-3′)

GAPDH(F:5′-CAGTGCCAGCCTCGTCTCAT-3′; R:5′-AGGGGCATCCACAGTCTTC-3′)

### 2.7. Western Blotting

Total protein was extracted from the heart tissues collected from the peri-infarcted area as well as H9C2 cells harvested at the end of the vitro experiments using radio-immunoprecipitation assay (RIPA) lysate buffer (Roche, CA, USA). The protein concentration was determined by the bicinchoninic acid protein quantitative method. The tissue/cell lysate samples were separated by 8–12% sodium dodecyl sulfate-polyacrylamide gel (SDS-PAGE) under 100 constant voltage for 1.5 h with electro-transferred (300 mA for 1.5 h at 4 °C) to nitrocellulose membranes (Millipore, MA, USA). The membranes were incubated with 3% BSA for 60 min at room temperature followed by incubation with one of the following primary antibodies overnight at 4 °C using the following dilution concentrations and commercial suppliers: FNDC5 (1:2000, ab174833, Abcam, MA, USA), PINK1 (1:1000, BS8731, Bioworld, MN, USA), Parkin (1:1000, ab179812, Abcam, MA, USA), LC3 (1:1000, ab128025, Abcam, MA, USA), P62 (1:1000, 18420-1-AP, Proteintech, IL, USA), OPA1 (1:1500, ab157457, Abcam, MA, USA), SOD2 (1:1000, GTX116093, GeneTex, CA, USA), AMPK (1:1000, D5A2, CST, MA, USA), p-AMPK (1:1000, 45F5, CST, MA, USA). GAPDH (1:5000) and β-tubulin (1:5000) were used as internal control for equal sample loading at 4 °C overnight. On the second day, the membranes were incubated with the HRP-conjugated secondary antibody at room temperature for 2 h. After the membranes were washed with TBST for three times, ECL liquid (Bio-Rad, CA, USA) was added to the membranes for label observation. Finally, protein bands were detected by Image Systems (Bio-Rad, CA, USA).

### 2.8. Statistical Analysis

Statistical analysis was conducted using GraphPad Prism 7.01 analysis software. Three independent experiments were made. Data were expressed as Mean ± Standard Error (SEM). One-way ANOVA was used for evaluating the significant differences of the mean values. ROUT test is used to detect outliers (outliers were not found). Histograms were post hoc test. Differences were considered statistically significant at * *p* < 0.05, ** *p* < 0.01.

## 3. Results

### 3.1. Different Types of Exercise and Skeletal Muscle Electrical Stimulation Intervention Enhanced Myocardial Irisin/FNDC5 Expression and Cardiac Function

**Figure 2:**
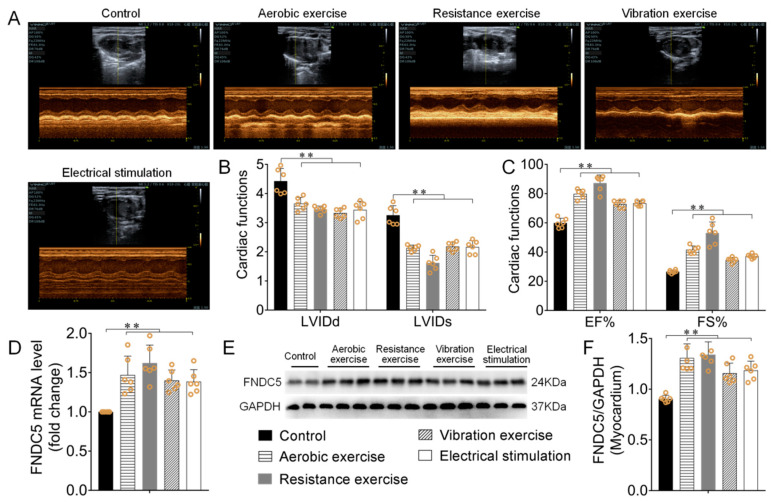
Different types of exercise and skeletal muscle electrical stimulation intervention enhanced myocardial irisin/FNDC5 expression and cardiac function. (**A**–**C**) The cardiac function of mice after different types of exercise and skeletal muscle electrical stimulation intervention. (**D**–**F**) The effect of mice’s myocardial *Irisin/FNDC5 mRNA* and its protein expression. Data are expressed as mean ± SEM (*n* = 6). ** *p* < 0.01. Abbreviations: FNDC5: transmembrane protein fibronectin type III domain protein 5, LVIDs: Left ventricular internal diameter at end systole, LVIDd: Left ventricular internal diameter at end diastole, EF: ejection fraction, FS: left ventricular fractional shortening.

In non-MI mice, different types of exercise and skeletal muscle electrical stimulation intervention significantly up-regulated myocardial *Irisin/FNDC5 mRNA* and its protein expression (*p* < 0.01), There is no striking difference between groups, while the effect of resistance exercise group is more significant (Figure 2D–F). Left ventricular internal diameter at the end systole (LVIDs), left ventricular internal diameter at end diastole (LVIDd), ejection fraction (EF), and left ventricular fractional shortening (FS) are commonly used to reflect cardiac systolic function [22]. Echocardiography results showed different types of exercise and skeletal muscle electrical stimulation markedly decreased myocardial LVIDd and LVIDs, increased EF and FS (*p* < 0.01, Figure 2A–C). These results showed that different types of exercise and skeletal muscle electrical stimulation significantly up-regulated myocardial irisin/FNDC5 expression, increased cardiac function. There is no striking difference between all groups, the effect of the resistance exercise group is more significant.

### 3.2. Different Types of Exercise and Skeletal Muscle Electrical Stimulation Intervention Activated the Myocardial PINK1/Parkin Pathway and Enhanced Antioxidant Function

**Figure 3:**
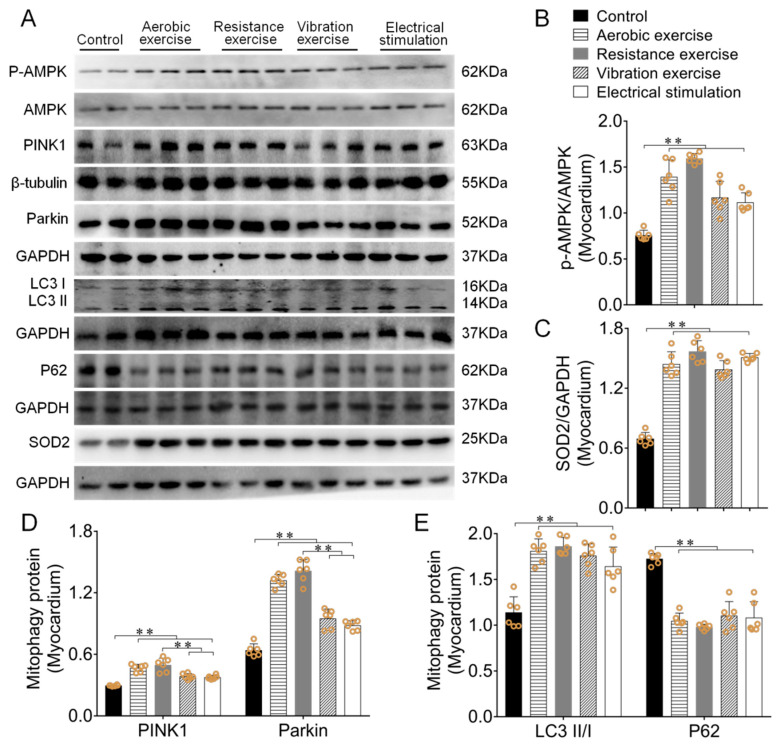
Different types of exercise and skeletal muscle electrical stimulation intervention activated myocardial mitophagy and enhanced antioxidant function. (**A**–**E**) The effect of myocardial PINK1, Parkin, P62, SOD2, LC3II/I and p-AMPK/AMPK ratio after different types of exercise and skeletal muscle electrical stimulation intervention. Data are expressed as mean ± SEM (*n* = 6). ** *p* < 0.01. Abbreviations: AMPK: Adenosine monophosphate (AMP)-activated protein kinase; PINK1: PTEN-induced kinase 1; Parkin: E3 ubiquitin ligase parkin; LC3II/I: microtubule-associated protein light chain 3; P62: SQSTM1/p62, SOD2: superoxide dismutase 2.

PINK1, Parkin, LC3 and P62 are the key proteins in mitophagy [23]. When mitochondrial damage occurs, phosphorylated PINK1/Parkin recruits P62 and binds to LC3 to maintain its function [12]. Western blotting results indicated that different types of exercise and skeletal muscle electrical stimulation intervention increased the phosphorylation level of AMPK (*p* < 0.01, Figure 3A,B), SOD2 expression in mice myocardium (*p* < 0.01, Figure 3A,C). Mitophagy related factors PINK1 and Parkin protein expressions were also significantly increased, and the effect of resistance exercise was more significant (*p* < 0.01, Figure 3A,D). In addition, different types of exercise and skeletal muscle electrical stimulation enhanced mice myocardial LC3II/I ratio (*p* < 0.01) and reduced P62 protein expression (*p* < 0.01, Figure 3A,E).

### 3.3. Different Types of Exercise and Skeletal Muscle Electrical Stimulation Intervention Activated the Mitochondrial PINK1/Parkin Pathway and Enhanced Antioxidant Function

**Figure 4:**
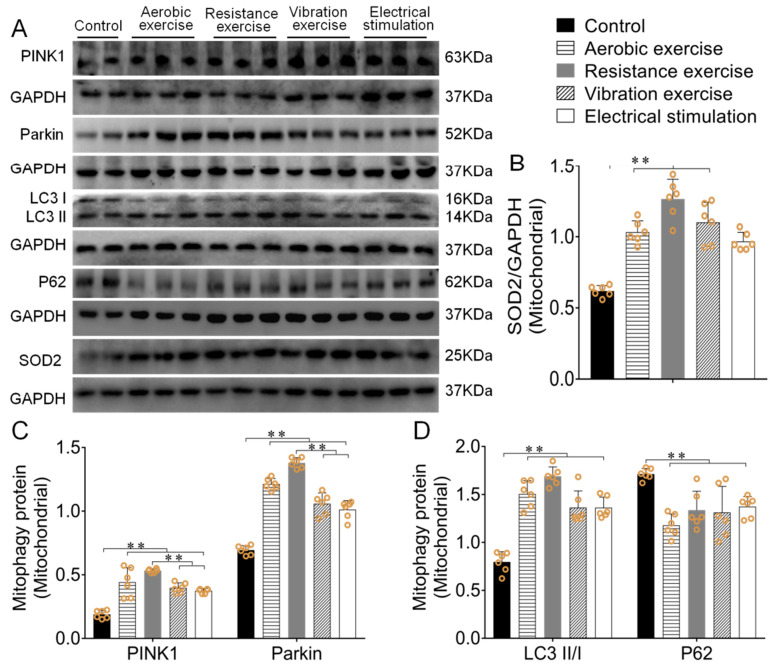
Different types of exercise and skeletal muscle electrical stimulation intervention activated mitophagy in mitochondria and enhanced antioxidant function. (**A**–**D**) The effect of mice’s myocardial mitochondrial PINK1, Parkin, P62, SOD2 and LC3II/I ratio after different types of exercise and skeletal muscle electrical stimulation intervention. Data are expressed as mean ± SEM (*n* = 6). ** *p* < 0.01. Abbreviations: PINK1: PTEN-induced kinase 1; Parkin: E3 ubiquitin ligase parkin; LC3II/I: microtubule-associated protein light chain 3; P62: SQSTM1/p62, SOD2: superoxide dismutase 2.

In order to reduce the other effective factors (the difference of membrane integrity or/and the openness of mitochondrial permeability transition pore) on mitophagy, external stimulation or other abnormal states can impair mitochondrial membrane integrity and lead to the abnormal opening of mitochondrial permeability transition pores, as well as leakage of mitochondrial cytokines (such as PINK1/Parkin, etc.) into the cytoplasm [24]. In order to reduce the influence of these factors on the level of mitophagy, mice myocardial mitochondrial protein was further extracted to verify the above results. Western blotting results showed the same trend of mitophagy-related proteins in PINK1/Parkin pathway and SOD2 expression both in the mitochondrial and myocardium (*p* < 0.01, Figure 4A–D). Among them, resistance exercise has the best effect.

### 3.4. Effect of Different Types of Exercise and Skeletal Muscle Electrical Stimulation Intervention on Myocardium L-OPA1/S-OPA1 Ratio and Its Correlation with Irisin/FNDC5

**Figure 5:**
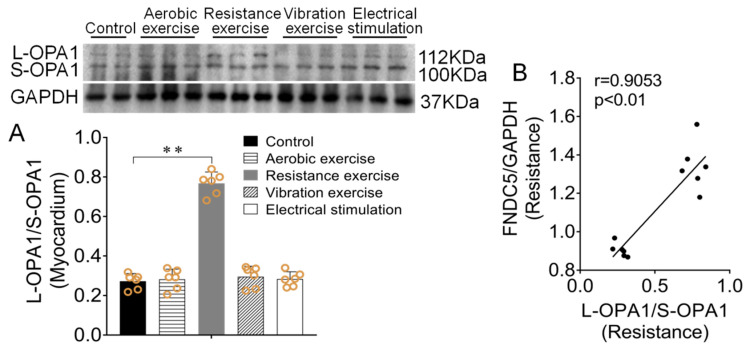
Effect of different types of exercise and skeletal muscle electrical stimulation intervention in mice myocardium OPA1 expression. (**A**) The effect of different types of exercise and skeletal muscle electrical stimulation intervention on mice’s myocardium L-OPA1/S-OPA1 ratio. (**B**) The Pearson correlation analysis between FNDC5 and OPA1 in myocardium after resistance exercise. Data are expressed as mean ± SEM (*n* = 6). ** *p* < 0.01. Abbreviations: FNDC5: transmembrane protein fibronectin type III domain protein 5, OPA1: Optic atrophy protein-1.

OPA1 mediates mitochondrial inner membrane fusion. There exist two forms: L-OPA1 and S-OPA1. L-OPA1 mediates mitochondrial fusion, while S-OPA1 inhibits mitochondrial fusion. The ratio of L-OPA1/S-OPA1 was often used to reflect the level of mitochondrial fusion. Western blotting results showed that mice myocardial L-OPA1/S-OPA1 ratio in resistance exercise group was significantly increased compared with the control group (*p* < 0.01, Figure 5A), Pearson correlation analysis was used to detect the correlation between OPA1 ratio and FNDC5 protein expression (Figure 2F). The results showed that myocardial L-OPA1/S-OPA1 ratio had clear positive correlation with irisin/FNDC5 protein expression in the resistance exercise (r = 0.9053, *p* < *0.01*, Figure 5B), and the other three intervention methods had no significant effect (r = 0.0896, *p* > 0.05; r = 0.4081, *p* > 0.05; r = 0.0288, *p* > 0.05).

### 3.5. AICAR Intervention Enhanced H9C2 Cells Irisin/FNDC5 Expression, Increased Mitophagy and Antioxidant Function

**Figure 6:**
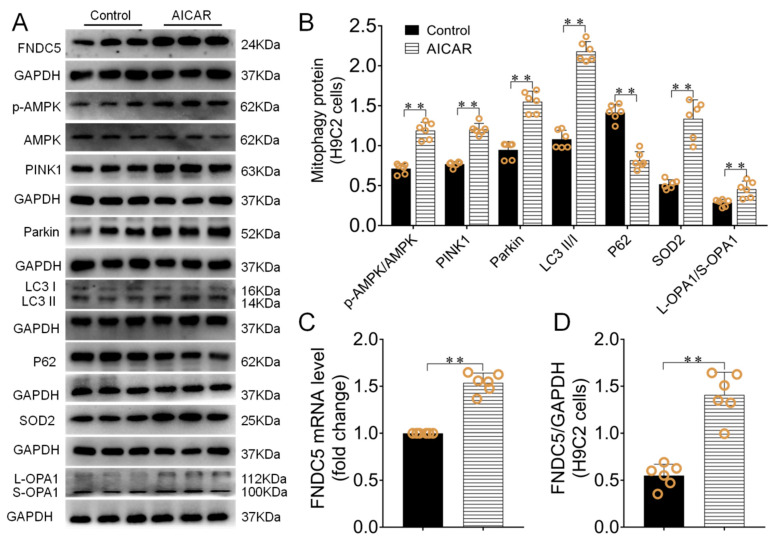
AICAR intervention enhanced irisin/FNDC5 expression, increased mitophagy and antioxidant function in H9C2 cells. (**A**,**B**) The effect of AICAR intervention on PINK1, Parkin, P62, SOD2 protein expressions and p-AMPK/AMPK, L-OPA1/S-OPA1 and LC3II/I ratio of H9C2 cells. (**C**) H9C2 cells *Irisin/FNDC5 mRNA* expression after AICAR intervention. (**D**) The effect of AICAR intervention on irisin/FNDC5 protein expressions. Data are expressed as mean ± SEM. ** *p* < 0.01. Abbreviations: FNDC5: transmembrane protein fibronectin type III domain protein 5; AMPK: adenosine monophosphate (AMP)-activated protein kinase; PINK1: PTEN-induced kinase 1; Parkin: E3 ubiquitin ligase parkin; LC3II/I: microtubule-associated protein light chain 3; P62: SQSTM1/p62, SOD2: superoxide dismutase 2; OPA1: optic atrophy protein-1.

AICAR was used to observe its influence on H9C2 cells irisin/FNDC5 expression, mitophagy of the PINK1/Parkin pathway and antioxidative capacity. The results displayed that *FNDC5 mRNA* and protein expressions were significantly enhanced in the AICAR group compared with the control group (*p* < 0.01, Figure 6A,C,D). H9C2 cells L-OPA1/S-OPA1 ratio also significantly enhanced after AICAR intervention (*p* < 0.01, Figure 6A,B), which had clear positive correlation with irisin/FNDC5 expression (r = 5929, *p* < 0.05). Besides, PINK1/Parkin expressions and LC3II/I ratio significantly increased (*p* < 0.01), P62 expression significantly decreased (*p* < 0.01, Figure 6A,B). In addition, the SOD2 protein expression was increased after AICAR intervention (*p* < 0.01, Figure 6A,B).

### 3.6. Resistance Exercise Enhanced MI Myocardial Irisin/FNDC5 Expression, Promoted Mitophagy, Enhanced Antioxidative Capability and Alleviated the Levels of Oxidative Stress

**Figure 7:**
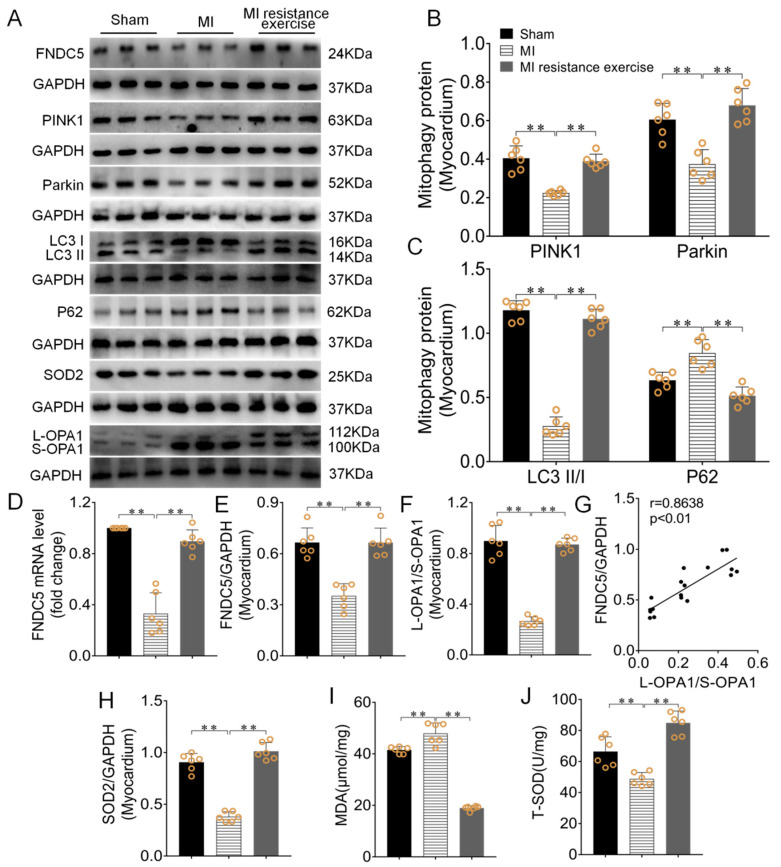
Resistance exercise enhanced MI myocardial irisin/FNDC5 expression, promoted mitophagy, enhanced antioxidative capability and alleviated the levels of oxidative stress. (**A**–**C**,**E**,**F**,**H**) The effect of resistance exercise on irisin/FNDC5, PINK, Parkin, P62, SOD2, protein expressions and L-OPA1/S-OPA1 and LC3II/I ratio in MI myocardium. (**D**) The effect of resistance exercise on *Irisin/FNDC5 mRNA*. (**G**) The Pearson correlation analysis between irisin/FNDC5 and OPA1 in MI myocardium. (**I**,**J**)The changes of T-SOD and MDA in MI and MI resistance exercise groups. Data are expressed as mean ± SEM (*n* = 6). ** *p* < 0.01. Abbreviations: FNDC5: transmembrane protein fibronectin type III domain protein 5, PINK1: PTEN-induced kinase 1, Parkin: E3 ubiquitin ligase parkin, LC3II/I: Microtubule-associated protein light chain 3, P62: SQSTM1/p62, OPA1: Optic atrophy protein-1, SOD2: Superoxide dismutase 2, MDA: Malondialdehyde, T-SOD: Total Superoxide dismutase.

Western blotting results showed that *Irisin/FNDC5 mRNA* and its protein expression was significantly decreased, PINK1/Parkin expressions and LC3II/I ratio significantly decreased in MI group (*p* < 0.01), while P62 expression clearly increased (*p* < 0.01) compared with the sham group. Resistance exercise training reverse these results (*p* < 0.01, Figure 7A–E). The irisin/FNDC5 expression had obvious positive correlation with EF (r = 5929, *p* < 0.05). It is found that the lower OPA1 level caused by MI can be reversed by resistance exercise training (*p* < 0.01, Figure 7A,F). The L-OPA1/S-OPA1 ratio had obvious positive correlation with irisin/FNDC5 expression (r = 0.8638, *p* < 0.01, Figure 7G).

Total superoxide dismutase (T-SOD) and malondialdehyde (MDA) are used to assess oxidative stress level [25]. The results of this study showed that MDA content induced by MI significantly enhanced, T-SOD and the protein expression of SOD2 were significantly reduced (*p* < 0.01). Resistance exercise training reversed these results. (*p* < 0.01, Figure 7A,H–J).

### 3.7. Resistance Exercise Improved MI Mice Cardiac Function

**Figure 8:**
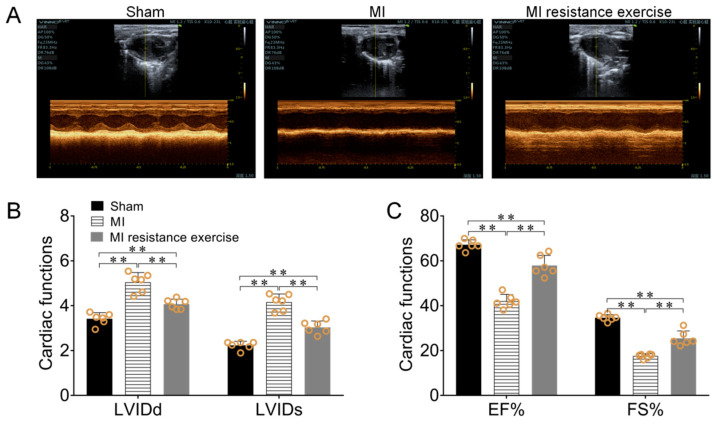
Resistance exercise reduced myocardial fibrosis, improved MI mice cardiac function. (**A**,**C**) Masson staining of heart sections for fibrosis evaluation and calculate fibrosis area by CVF. Collagen was stained blue and cardiomyocyte was stained red. (**A**–**C**) the effect of resistance exercise training on MI mice LVIDd, LVIDs, EF and FS. Data are expressed as mean ± SEM (*n* = 6). ** *p* < 0.01. Abbreviations: LVIDs: Left ventricular internal diameter at end systole, LVIDd: Left ventricular internal diameter at end diastole, EF: Ejection fraction, FS: Left ventricular fractional shortening.

Echocardiography results showed myocardial LVIDd and LVIDs increased (*p* < 0.01), EF and FS decreased (*p* < 0.01) in MI group. After resistance exercise intervention, LVIDd and LVIDs decreased (*p* < 0.01, Figure 8A,B), EF and FS increased (*p* < 0.01, Figure 8A,C). These results indicated that resistance exercise training improved MI cardiac function.

## 4. Discussion

Exercise involves protecting impaired cardiac function caused by MI, but the exact mechanism is not clear yet. The present study showed that different types of exercise and skeletal muscle electrical stimulation intervention enhanced irisin/FNDC5 expression, activated PINK1/Parkin pathway mitophagy, enhanced antioxidant function and improved cardiac function, and the effect of resistance exercise is more significant. The mice myocardial OPA1 expression in resistance exercise group significantly increased, which had clear positive correlation with irisin/FNDC5 expression, while the other three intervention methods had no significant effect. In addition, resistance exercise also significantly increased MI myocardial irisin/FNDC5 and OPA1 expression, enhanced PINK1/Parkin pathway mitophagy, alleviated oxidative stress and improved MI cardiac function. These results suggested that resistance exercise inhibited oxidative stress and improved cardiac function in MI, partially via activating FNDC5/Irisin-PINK1/Pakin-LC3II/I-P62 signaling pathway. OPA1 may play a major role in this process.

Irisin is a novel myokine and adipokine in various body tissues, including skeletal muscle, myocardium and adipose tissue, which can be effectively induced by several kinds of stimulation such as exercise training [9,14]. Some research showed that many different types of exercise can significantly increase the myocardial Irisin expression [26,27,28,29]. This study found that the expression of myocardium irisin/FNDC5 was significantly up-regulated in different exercise intervention and skeletal muscle electrical stimulation models, among which the resistance exercise had the significant effects. Previous studies demonstrated that irisin/FNDC5 significantly inhibited cardiomyopathic hypertrophy, reduced cell apoptosis and alleviated myocardial fibrosis, and ultimately improved cardiac function in MI model [30,31,32]. Our present study confirmed that MI significantly decreased the irisin/FNDC5 expression in the myocardium. Resistance exercise training significantly up-regulated irisin/FNDC5 expression, improved the cardiac function in mice. Previous studies indicated that resistance exercise training stimulated the secretion of skeletal muscle-associated proteins and significantly promoted the skeletal muscle growth [33]. Skeletal muscle-derived irisin can be transported to the lungs via circulation, which plays an important role in the improvement of lung injury caused by ischemia/reperfusion [34]. Therefore, it can be assumed that resistance exercise training might improve cardiac function in MI mice due to higher secretion of Irisin.

Autophagy is an important mechanism to maintain cell homeostasis, which can be divided into selective and non-selective pathway [35]. Mitophagy is a kind of selective autophagy which plays an indispensable role in maintaining cellular homeostasis under several conditions, and the PINK1/Parkin pathway is its main route [36]. When mitochondria function is impaired, PINK1 increased in outer mitochondrial membrane and combined with the transferring enzyme to promote itself phosphorylation, then recruited the activated cytoplasmic Parkin, and further recruited P62 to combine with LC3, prompted the damaged mitochondria to be degraded, eventually completed mitophagy [37]. PINK1 and Parkin expression could significantly decrease in the *Irisin/FNDC5* knockout mice and accompanied with mitochondrial dysfunction in myocardium [38,39], which suggested that irisin/FNDC5 was positively correlated with PINK1 and Parkin expression. Our present study provided direct evidence for exercise induced up-regulated expression of Irisin and enhanced PINK1/Parkin-mediated mitophagy in the myocardium, which was consistent with the results of previous studies [40,41,42]. Our study also presented that resistance exercise group had better effect in different types of exercise and skeletal muscle electrical stimulation intervention. In addition, we further tested myocardial mitochondrial protein to verify the above results, which can reduce the other effective factors on mitophagy. The same trend of mitophagy-related proteins in PINK1/Parkin pathway expression was found both in the mitochondrial and in the myocardium, while the resistance exercise had better effect.

The previous studies demonstrated that MI led to SOD inactivity, increased MDA and reactive oxygen species (ROS) accumulation, and broke the balance of oxidation and antioxidant [43,44,45]. Exercise intervention can significantly up-regulate the expression of Irisin and SOD activity in the heart, liver, kidney and other organs of mice under the circumstances of ischemia and hypoxia, while reduce the level of MDA and ROS [46,47,48]. Irisin contributed to the maintaining oxidative balance in organs and tissues [49]. It has been reported that both swimming and treadmill protocol enhanced the Irisin expression in MI rat’s serum [50,51]. Similarly, previous studies in our laboratory confirmed that aerobic exercise can significantly increase the Irisin expression in the kidney of MI mice and simultaneously inhibit the oxidative stress [48]. Our current results showed that for MI mice, the resistance exercise significantly enhanced the expression of Irisin, increased the activity of T-SOD and SOD2 expression, decreased the content of serum MDA, and improved the level of oxidative stress in myocardium.

Recent research showed that OPA1 played a key role in enhancing myocardial mitophagy and improving cardiac function [40,41]. Different forms of exercise intervention could activate the OPA1 expression in the myocardium [9,52]. Meanwhile, Irisin activated mitophagy in MI cardiomyocytes induced by OPA1 [38,53,54]. Taken together, we focused on the role of OPA1 and Irisin in resistance exercise-induced cardio protection in the MI myocardium. This study compared the expression of OPA1 in myocardium after different kinds of exercise and skeletal muscle electrical stimulation intervention, and it was shown that resistance exercise can significantly up-regulate the expression of OPA1 in myocardium, which was correlated with the expression of Irisin positively. AICAR intervention to simulate exercise also significantly increased the expression of OPA1 in H9C2 cells. Furthermore, the results suggested the OPA1 expression trend was consistent with irisin/FNDC5 both in vitro and in vivo experiments. According to the above results, there possibly exists a speculated regulatory relationship among Irisin, OPA1 and mitophagy, which is affected by the forms of exercise intervention. Several studies have shown that exercise significantly increases AMPK phosphorylation, but the level of improvement may depend on exercise load [55]. The expression of OPA1 significantly increased in long-term exercise with moderate to high intensity, whereas exercise with acute or low intensity had no significant effect [52,54,56]. Therefore, we speculate that OPA1 expression may be affected by the phosphorylation of AMPK, but the potential mechanism remains to be further explored.

AMPK is a key energy sensor that regulates cell metabolism to maintain energy balance, and is closely related to mitophagy [57,58]. Inhibition of AMPK expression can lead to abnormal mitophagy, aggravate oxidative stress and apoptosis levels in the myocardium [59,60]. oleuropein, tilianin, metformin and AICAR have been shown to exert cardioprotective effects as AMPK agonists [61,62,63,64,65]. For example, oleuropein has a strong anti-inflammatory and antioxidant capacity, it can reduce myocardial infarction area and protect cardiac function by upregulating the expression of myocardial AMPK [61,62]. AICAR proved to play a cardioprotective role as an AMPK agonist in a variety of disease models (e.g., hypertensive [65], acute kidney injury [66], cardiac hypoxia [67]). The previous research also used AICAR as AMPK agonist to explore the protective effect of exercise on ischemic myocardium [44,48]. This study showed that AICAR could significantly up-regulate the expression of AMPK in H9C2 cells, activate the PINK1/Parkin-LC3/P62 signaling pathway, and enhance mitophagy.

This present study has several limitations: (1) Due to the wide variety of exercise parameters such as exercise modes, exercise intensity, exercise duration, etc., this experiment has not been fully involved. The corresponding intervention forms are based on the preliminary research of this laboratory and literature reports. (2) It has been well proved that exercise can activate the PINK1/Parkin-LC3/P62 pathway and regulate mitophagy level. This study lacks of endogenous intervention (irisin knockin or knockout) to further explore the irisin-mediated PINK1/Parkin-LC3/P62 mitophagy signaling pathway activated by exercise. (3) mPTP transition is an end-target of cardioprotection [68]. However, due to the difficulty of modeling, long cycle and small number of experimental samples in this study, the detection of mitochondrial function and mPTP transition was missing in this study. (4) Currently, many kinds of experimental methods can be used to detect mitophagy [69], this study lacks in verifying the changes of mitophagy from other perspectives.

## 5. Conclusions

**Figure 9:**
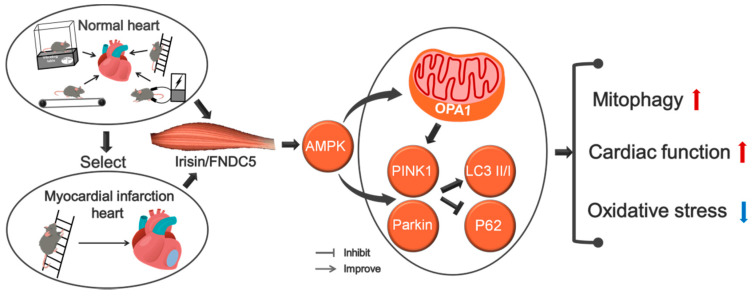
Resistance exercise mediated by Irisin enhanced mitophagy and improved cardiac function.

Different types of exercise and skeletal muscle electrical stimulation can increase the expression of myocardial irisin/FNDC5, promote mitophagy and improve cardiac function in the normal mice, and resistance exercise has the most significant effects. Resistance exercise significantly inhibits oxidative stress, regulates mitophagy and improves cardiac function in MI mice via activating myocardial irisin/FNDC5-PINK1/Parkin-LC3/P62 pathway, OPA1 may plays an important role in this series of protective effects (Figure 9).

## Data Availability

Data sharing not applicable. The data are not publicly available due to privacy restriction.

## Abbreviations

The following abbreviations are used in this manuscript:

AICAR	5-amino-1-β-d-ribofuranosyl-imidazole-4-carboxamide
AMPK	Adenosine monophosphate (AMP)-activated protein kinase
ANOVA	Analysis of variance
EF	Ejection fraction
FNDC5	Fibronectin type III domain protein 5
FS	Fractional shortening
LAD	Left anterior descending coronary artery
LC3	Microtubule-associated protein light chain 3
LVIDd	Left ventricle internal dimension diastole
LVIDs	Left ventricle internal dimension systole
MI	Myocardial infarction
MDA	Malondialdehyde
OPA1	Optic atrophy protein-1
P62	SQSTM1/p62
Parkin	E3 ubiquitin ligase parkin
PINK1	PTEN-induced kinase 1
RIPA	Radio-immunoprecipitation assay
ROS	Reactive oxygen species
SDS-PAGE	Sodium dodecyl sulfate-polyacrylamide gel
SOD2	Superoxide dismutase 2
T-SOD	Total Superoxide dismutase
WT	Wildtype
